# Effect of Coffee Consumption on the Risk of Gastric Cancer: A Systematic Review and Meta-Analysis of Prospective Cohort Studies

**DOI:** 10.1371/journal.pone.0128501

**Published:** 2015-05-29

**Authors:** Haibin Liu, Ying Hua, Xiangyun Zheng, Zhaojun Shen, Hui Luo, Xuejiao Tao, Zhiyi Wang

**Affiliations:** 1 Department of General Surgery, The Second Affiliated Hospital of Wenzhou Medical University, Wenzhou 325027, China; 2 Department of Obstetrics and Gynecology, The Second Affiliated Hospital of Wenzhou Medical University, Wenzhou 325027, China; 3 Department of Emergency Medicine, The Second Affiliated Hospital of Wenzhou Medical University, Wenzhou 325027, China; University Hospital Llandough, UNITED KINGDOM

## Abstract

**Background and Objectives:**

Results from observational epidemiologic studies on the relationship between coffee consumption and gastric cancer are inconsistent and inconclusive. To assess the association between coffee consumption and the risk of gastric cancer, we summarized evidence from prospective cohort studies.

**Methods:**

Relevant studies were retrieved through computer searches (PubMed, EmBase and the Cochrane Library) and a review of references up to December 2014. The quality of the included studies was evaluated by Newcastle-Ottawa quality assessment scale. We used a meta-analytic approach to estimate overall hazard ratios (HRs) and 95% confidence intervals (CIs) for regular coffee drinkers versus individuals who seldom drank coffee. Sensitivity analysis and subgroup analysis were performed to assess the reliability of our results. A dose–response analysis was performed to assess the risk of gastric cancer based on the level of coffee consumption.

**Results:**

Nine prospective cohort studies involving 1,250,825 participants and 3027 gastric cancer cases were included in this meta-analysis. The pooled HR of gastric cancer for the study-specific regularly versus seldom coffee drinking categories was 1.05 (95% CI, 0.88 to 1.25) with significant heterogeneity across studies (I^2^ = 74.0%, *P* = 0.000). After the sensitivity analysis, three studies were deleted; however the association remained insignificant (HR, 0.99; 95% CI, 0.91 to 1.08). Subgroup analysis by anatomic location showed a risk for coffee consumption associated with cardia cancer (HR, 1.23; 95% CI, 1.04 to 1.45; heterogeneity, I^2^ = 36.4, *P* = 0.207). In the dose–response analysis, there was no significant association between coffee intake (in cups) and the risk of gastric cancer (*P* for linearity trend and non-linearity > 0.05).

**Conclusion:**

Our meta-analysis demonstrated that coffee consumption was not associated with overall gastric cancer risk; however, coffee consumption may be a risk factor for gastric cardia cancer.

## Introduction

Gastric cancer is the fourth most common cancer in men and the fifth most common cancer in women worldwide [1]. An estimated 951,600 people suffered from gastric cancer and 723,100 gastric cancer cases died in 2012 [1]. The incidence of gastric cancer varies considerably based on geographical location [2], which suggested that ethnic group, environmental exposures, socioeconomic status and lifestyle factors were associated with these regional differences [1]. Dietary factor is one of lifestyle factors and it can explain these differences [3].

Coffee is one of the most popular beverages consumed worldwide. The relationships between coffee and various types of cancer have been studied for many years. Human experimental studies on such relationships are unlikely, making observational studies the best available source of evidence to evaluate the risk. Since the 1960s, several observational epidemiological studies have investigated the relationship between coffee consumption and gastric cancer, but the findings were inconsistent. Thus, clarifying the association between coffee consumption and the risk of gastric cancer has important public health implications.

A recent meta-analysis has suggested that there was no statistically significant association between coffee consumption (highest vs. lowest consumption) and the risk of gastric cancer [4]. However, the meta-analysis included studies with different outcome measures (morbidity or mortality) and pooled studies with different coffee consumption categories. Those misclassifications of outcome measures and coffee consumption categories might have influenced the results of their meta-analysis, and made their conclusions have been questioned. In an attempt to further elucidate the association between coffee consumption and the risk of gastric cancer, we conducted a systematic review and meta-analysis of prospective cohort studies. To quantitatively assess the effects of coffee consumption on gastric cancer, we also conducted a dose-response analysis.

## Methods

### Literature search strategies

We searched PubMed, EmBase and the Cochrane Library to identify relevant studies before December 2014. Taking PubMed search string as an example, the following search strategy was carried out: #1 Coffee [MH] OR coffee [TIAB], #2 "Stomach Neoplasms" [MH] OR ((gastric [TIAB] OR stomach [TIAB] OR cardia [TIAB]) AND (cancer* [TIAB] OR carcinoma* [TIAB])), #3 #1 AND #2. There was no restrictions regarding language, type of publication and publication status. Furthermore, we reviewed citations from the retrieved articles and relevant reviews to identify additional studies. This systematic review was planned, conducted and reported in accordance with the meta-analysis quality standards of observational studies [5].

### Eligibility criteria

Duplicate and irrelevant articles were excluded based on the title and abstract. Full-text articles were later screened for eligibility. Our meta-analysis included only prospective cohort studies that reported the effect of coffee consumption on the risk of gastric or cardia cancer. The outcome measure was the incidence of gastric or cardia cancer. All included studies provided the effect size (relative risk [RR] or hazard ratio [HR]) and the corresponding 95% confidence intervals (CI), or provided enough data to calculate them. When several papers from the same study had been published, only the most complete or most recent paper was used. All potentially relevant studies were independently screened for eligibility by two authors. Discrepancies between the authors were resolved through discussion.

### Data extraction and study quality assessment

We did not attempt to obtain additional information from the authors of the original studies. The following information was extracted from each study: the last name of first author, publication year, country of residence, specific study groups, sex, follow-up time, number of cases, number of participants, categories of coffee consumption, HR or RR estimates for each level of coffee consumption and the corresponding 95% CIs, and confounding factors adjusted for in the analysis. When a study did not report the effect size (ES) and the corresponding 95% CI, we calculated them using the raw data. If a study provided more than one ES, the ES that adjusted for the largest number of confounding factors was included in the analysis. The Newcastle-Ottawa quality assessment scale (NOS) [6] was used to assess the quality of the included studies. A high-quality study was defined as a study with 7 or more stars. Data were independently extracted by two of the authors. Conflicting evaluations were resolved either through discussion or third party resolution.

### Statistical analysis

We used HRs and the corresponding 95% CIs to measure the association between coffee consumption and the risk of gastric cancer. The RRs were deemed to be equivalent to HRs. In any included study, when the effect sizes were reported separately for the subgroups based on the different levels of coffee consumption, we combined the subgroup results and used a fixed-effects model to calculate a common effect size for the main analysis. The “seldom drink coffee” category was defined as “do not drink coffee every day, never drink coffee, less than or equal to 1 cup, once, or 131ml per day”, whereas other consumption levels were identified as “regular coffee drinker”. When a study reported HRs for cardia cancer and gastric non-cardia cancer separately, we also pooled the results using fixed-effects model. The data from individual studies were pooled utilizing the random-effects model (DerSimonian and Laird method) [7] when significant heterogeneity was observed among the studies. Otherwise, a fixed-effects model (Mantel-Haenszel method) [8] was applied.

Statistical heterogeneity among the studies was evaluated using the Q statistic and was considered to be statistically significant at *P* < 0.10 [9]. The *I* ^2^ statistic measures the percentage of total variation across studies because of heterogeneity rather than chance [10]. Heterogeneity was considered to be significant when the I^2^ statistic was ≥ 50%. In addition, summary estimates were calculated in specific subgroups.

A sensitivity analysis was conducted to evaluate the stability of the results and to explore the possible explanations for heterogeneity. Random-effects model was used for the above sensitivity analysis. In addition, subgroup analyses stratified by sex, anatomic location, duration of follow-up, place of residence, and adjustment for confounders were also performed to explore potential sources of heterogeneity.

A dose-response analysis was performed to describe the relationship between the coffee intake level and the risk of gastric cancer. A linear model proposed by Greenland and Longnecker [11] was used to assess the correlation between categories. Non-linear analysis was performed by fitting a class of two-term fractional polynomial models to the data [12], which was used to determine correlations among the reported estimates in the same study, the heterogeneity between studies, and the non-linear trend component of the dose-response relationship. For the dose-response analysis, we included studies that reported at least three coffee consumption levels and provided the number of cases and participants for each exposure category. Because coffee consumption is often presented as a range, we assigned the exposure value as the midpoint between the upper and lower boundaries for each coffee consumption category. If the upper boundary for the highest category was not provided, then the open-ended upper category was considered to be the same amplitude as the previous category, or the approximate midpoint of the highest category was defined as 1.5 times the lower boundary of that category. If the lower boundary for the lowest category was not provided, then the midpoint of the lowest category was assigned as half of the upper boundary of that category.

Publication bias was assessed with the Egger's test and the Begg's funnel plot, and the *P* value less than 0.05 was considered statistical significance. All above statistical analyses were conducted using STATA version 12.0 (Stata Corporation, College Station, TX, USA).

## Results

### Study identification and selection

**Fig 1:**
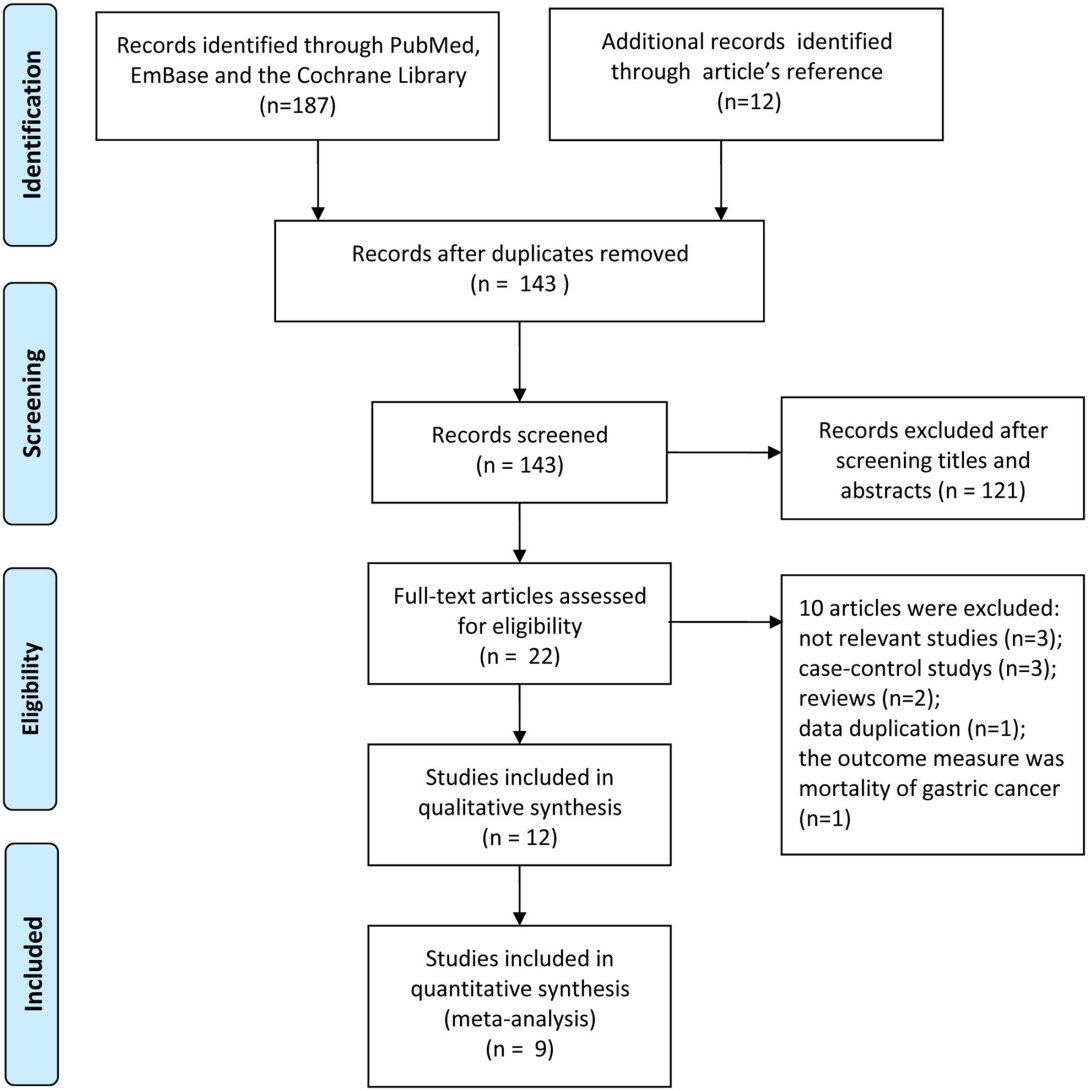
A flowchart identifying the studies that were included in the meta-analysis.

The workflow and results of the literature review are shown in Fig 1. Initially, we retrieved 187 potentially relevant studies from PubMed, EmBase and the Cochrane Library (82, 104 and 1 results, respectively) and identified 12 additional relevant articles after reviewing the reference lists in those studies. After 56 duplicates were excluded, the titles and abstracts of 143 articles were assessed, of which 121 articles were excluded because they did not meet the inclusion criteria. Subsequently, we reviewed the full texts of the remaining 22 potentially relevant articles. Ten articles were excluded for the following reasons: not relevant (n = 3); case-control study design (n = 3); reviews (n = 2); duplicate reports from the same study population (n = 1); the outcome measure was not the incidence of gastric cancer (n = 1). Finally, 12 studies [13–24] were included in the systematic review.

### Study characteristics and quality assessment

The main characteristics of the included studies are summarized in Table 1. Three studies were excluded from the final meta-analysis, because their reference categories were inconsistent with the reference categories for the meta-analysis. Nine studies remained for quantitative data synthesis were published between 1986 and 2014, included 3027 cases of gastric cancer and 1250825 participants; the number of participants per study ranged from 7355 to 480542. Among the studies, four were from Europe, two were from Asia, and three were from United States. There were two studies based in the United States, but the research populations were Japanese. The follow-up periods ranged from 6 to 18 years, and the average duration was 13.3 years. Possible confounding factors were adjusted for in 9 studies, of which 1 study adjusted for age only, and the other studies adjusted for sex, age, smoking, alcohol consumption, etc. The included studies achieved relatively high scores on the quality assessment (7–9 in total). The study-specific quality scores are summarized in Table 1.

**Table 1:** Main characteristics of included studies.

Author/							
Year/							
Country/			No. of cases/		HR or RR		
Special annotation/	Sex	Follow-up	No. of participants	Coffee consumption	(95%CI)	Study quality*	Confounders adjusted for
Nine studies included in the meta-analysis.					**HR(95%CI)**		
Galanis et al [13]	Both	14.8 years	In men	**0 cup/day**	**1.00(1.00–1.00)**	8	Japanese place of birth, age,
1998			64/5610	1 cup/day	2.50(1.00–6.10)		sex in combined analyses,
United States				≥2 cups/day	2.20(0.90–5.30)		smoking in men analyses,
Japanese in Hawaii			In women	**0 cup/day**	**1.00(1.00–1.00)**		alcohol intake in men analyses
			44/6297	1 cup/day	1.30(0.60–3.10)		education.
				≥2 cups/day	1.60(0.70–3.80)		
			In all	**0 cup/day**	**1.00(1.00–1.00)**		
			108/11907	1 cup/day	1.80(1.00–3.20)		
				≥2 cups/day	1.80(1.00–3.30)		
Larsson et al [14]	Women	15.7 years	160/61433	**≤1 cup/day**	**1.00(1.00–1.00)**	8	Age, alcohol intake,
2006				2-3cups/day	1.54(0.99–2.39)		tea consumption, education,
Sweden				≥4 cups/day	1.86(1.04–3.34)		time period.
Nilsson et al [15]	Both	15 years	151/64603	**<1 occasion/day**	≥4 occasions/day	7	Age, sex, BMI, smoking,
2010				1–3 occasions/day	0.66(0.31–1.43)		education, physical activity.
Sweden				≥4 occasions/day	0.99(0.44–2.21)		
Ren et al [16]	Both	6 years	6 years	**Gastric cardia**		8	Age, sex, smoking, BMI,
2010			454/480542	**<1 cup/day**	**1.00(1.00–1.00)**		alcohol drinking, education,
United States				= 1 cup/day	1.13(0.71–1.78)		ethnicity, physical activity,
				2-3cups/day	1.24(0.86–1.79)		vegetables, fruit, red meat,
				>3 cups/day	1.57(1.03–2.39)		white meat and calories.
				**Non-cardia**			
				**<1 cup/day**	**1.00(1.00–1.00)**		
				= 1 cup/day	0.96(0.63–1.47)		
				2-3cups/day	1.07(0.76–1.52)		
				>3 cups/day	1.06(0.68–1.64)		
Bidel et al [17]	Both	18 years	In men	**0 cup/day**	**1.00(1.00–1.00)**	9	Age, education, study year,
2013			181/29159	1–2 cups/day	0.78(0.40–1.51)		sex, alcohol consumption,
Finland				3–4 cups/day	0.51(0.27–0.92)		smoking, physical activity,
				5–6 cups/day	0.50(0.27–0.92)		tea consumption, diabetes,
				7–9 cups/day	0.54(0.28–1.06)		BMI.
				≥10 cups/day	0.53(0.26–1.09)		
			In women	**0 cup/day**	**1.00(1.00–1.00)**		
			118/30882	1–2 cups/day	1.87(0.54–6.52)		
				3–4 cups/day	1.41(0.42–4.69)		
				5–6 cups/day	1.35(0.40–4.49)		
				7–9 cups/day	1.33(0.37–4.87)		
				≥10 cups/day	2.07(0.53–8.15)		
			In all	**0 cup/day**	**1.00(1.00–1.00)**		
			299/60041	1–2 cups/day	0.94(0.53–1.65)		
				3–4 cups/day	0.64(0.37–1.09)		
				5–6 cups/day	0.62(0.36–1.05)		
				7–9 cups/day	0.67(0.37–1.20)		
				≥10 cups/day	0.75(0.40–1.41)		
Ainslie-Waldman et al [18]	Both	14.7 years	In men	**Nondaily**	**1.00(1.00–1.00)**	9	Age, sex, smoking,education,
2014			394/27293	Daily	1.03(0.79–1.34)		interview year, BMI, dialect,
Singapore			In women	**Nondaily**	**1.00(1.00–1.00)**		number of cigarettes per day,
Chinese in Singapore			253/34028	Daily	0.63(0.46–0.87)		years smoked, caffeine,
			In all	**Never/monthly**	**1.00(1.00–1.00)**		total energy intake.
			647/61321	1 cup/day	0.84(0.66–1.07)		
				2–3 cups/day	1.00(0.71–1.40)		
				≥4 cups/day	0.93(0.49–1.79)		
				**Nondaily**	**1.00(1.00–1.00)**		
				Daily	0.85(0.69–1.04)		
			Gastric cardia	**Nondaily**	**1.00(1.00–1.00)**		
				Daily	0.78(0.46–1.33)		
			Non-cardia	**Nondaily**	**1.00(1.00–1.00)**		
				Daily	0.68(0.46–1.01)		
Sanikini et al [19]	Both	11.6 years	In all	**Gastric cardia**		9	age, sex, center, intake of
2014			683/477312	**Non/Quartile 1**	**1.00(1.00–1.00)**		energy, smoking, education,
Ten European countries				Quartile 2	1.06 (0.65–1.72)		physical activity, diabetes,
				Quartile 3	1.41 (0.87–2.27)		BMI, alcohol consumption,
				Quartile 4	1.41 (0.86–2.30)		vegetable, fiber, fruit, fish,
				**Non-cardia**			red and processed meat.
				**Non/Quartile 1**	**1.00(1.00–1.00)**		
				Quartile 2	0.78 (0.56–1.08)		
				Quartile 3	0.90 (0.61–1.32)		
				Quartile 4	0.94 (0.63–1.40)		
					**RR(95%CI)**		
Nomura et al [20]	Men	15 years	In men	**0 cup/day**	**1.00(1.00–1.00)**	7	Age
1986			106/7355	1-2cups/day	1.32(0.71–2.42)^#^		
United States				3-4cups/day	1.70(0.93–3.11)^#^		
Japanese in Hawaii				≥5cups/day	1.18(0.62–2.25)^#^		
				**0 cup/day**	**1.00(1.00–1.00)**		
				≥1cups/day	1.40(0.80–2.43)		
Tsubono et al [21]	Both	9 years	419/26311	**never**	**1.00(1.00–1.00)**	9	Age, sex, smoking, tea,
2001				1-2cups/day	0.80(0.50–1.10)		consumption of alcohol, rice,
Japan				≥3 cups/day	1.00(0.60–1.60)		meat, vegetables, fruits,
							bean-past soup,
							type of health insurance.
Three studies excluded from the meta-analysis.					**RR(95%CI)**		
Jacobsen et al [22]	Both	11.5 years	147/16555	**≤2 cups/day**	**1.00(1.00–1.00)**	6	Age, sex, residence.
1986				≥7 cups/day	1.46(0.84–2.55)^#^		
Norway							
Stensvold & Jacobsen [23]	Both	11.1 years	In men	**≤2 cups/day**	**1.00(1.00–1.00)**	9	Age, smoking,
1994			46/21735	≥7 cups/day	0.68(0.28–1.69)^#^		county of residence.
Norway			In women	**≤2 cups/day**	**1.00(1.00–1.00)**		
			32/21238	≥7 cups/day	0.47(0.16–1.39)^#^		
van Loon et al [24]	Men	4.3 years	In men	**≤3 cups/day**	**1.00(1.00–1.00)**	5	No
1998			146/1525	>4 cups/day	1.50(1.03–2.20)^#^		
Netherland							

*Study quality was judged on the basis of the Newcastle-Ottawa Scale (1–9 stars).

^#^estimated using data available in the article.

In the study by Sanikini et al, cohort-wide quartiles for levels of coffee consumption were computed after excluding non-consumers, and cut-off points (ml) for coffee quartiles were 131, 310 and 556.

### Main analysis, subgroup analysis and sensitivity analysis

**Fig 2:**
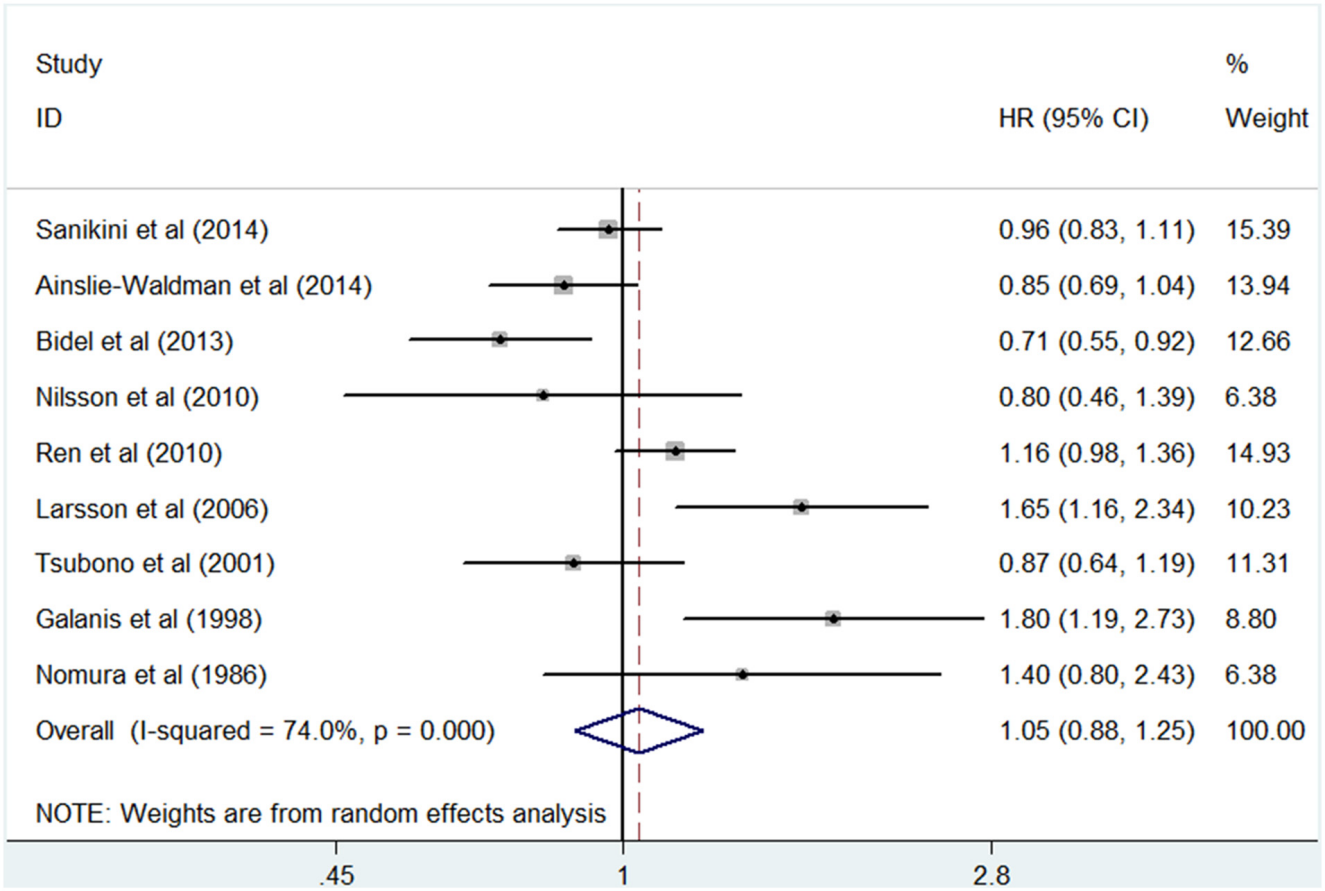
Forest plot of the 9 studies included in the meta analysis. A forest plot for the study-specific regularly versus seldom coffee drinking categories, showing the association between coffee consumption and the risk of gastric cancer.

**Fig 3:**
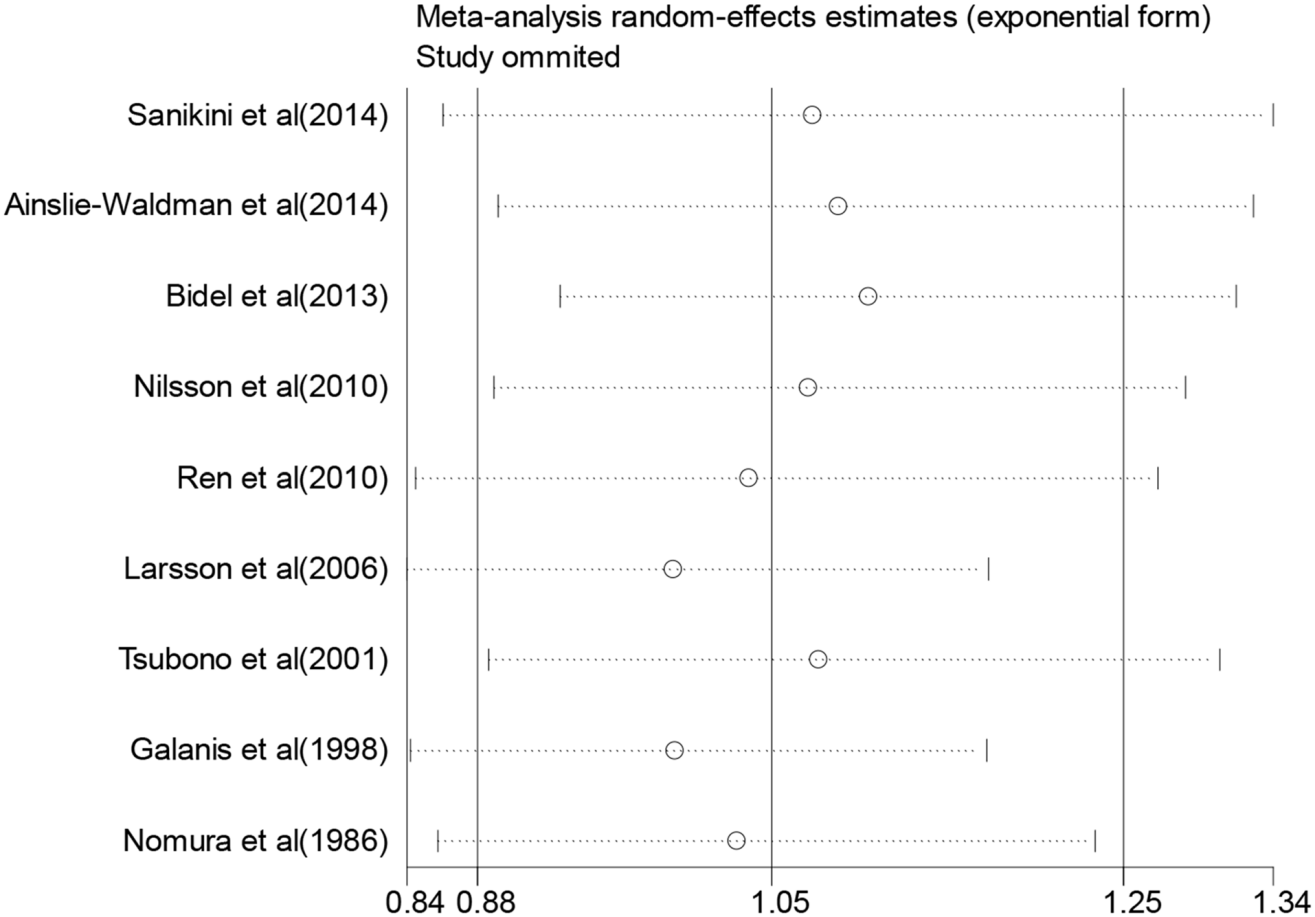
Influence analysis of the summary HRs for coffee consumption on gastric cancer risk. The meta-analysis random-effects estimates (exponential form) were used. The results were computed by omitting each study (on the left) in turn. The two ends of every broken line represent the 95% CIs.

For “regular coffee drinkers” vs. “seldom coffee drinkers”, 9 studies were included in the meta–analysis and used to summarize the HR estimates (Fig 2). The summary HR was 1.05 (95% CI, 0.88 to 1.25) with statistically significant heterogeneity among studies (I^2^ = 74.0%, *P* = 0.000). The sensitivity analyses indicated that any single study was not the main origin of heterogeneity among studies (Fig 3). Then we excluded any two or three studies in turn and pooled the data of the remaining studies. The heterogeneity was decreased (I^2^ = 57.6%, *P* = 0.028) after two studies by Galanis et al. [13] and Larsson et al. [14] were excluded, and was more effectively decreased (I^2^ = 40.4, *P* = 0.136) after the third study by Bidel et al. [17] was excluded (S1 Fig), however the association remained insignificant (HR, 0.93; 95% CI, 0.81 to 1.08, and HR, 0.99; 95% CI, 0.91 to 1.08, respectively). For those excluded studies, no unifying factor were identified as a possible source of heterogeneity among all studies. To further explore the reasons for the heterogeneity, we performed subgroup analyses according to sex, anatomic location, duration of follow-up, place of residence, race, or adjustment for confounders (Table 2). As shown in Table 2, most subgroup analyses showed no statistically significant association with significant heterogeneity between coffee and the risk of gastric cancer. In the subgroups according to place of residence, Asia and USA presented low heterogeneity (I^2^ = 0.0 and I^2^ = 49.2, respectively), and the pooled result from studies of USA showed a significant positive association (HR, 1.35; 95% CI, 1.02 to 1.81). The subgroup analysis by adjusted confounders, including smoking, alcohol drinking and dietary factors, also presented low heterogeneity (I^2^ = 47.5, *P* = 0.149), but the association remained insignificant (HR, 1.02; 95% CI, 0.87 to 1.18). What is remarkable, however, is that subgroup analysis by anatomic location showed a risk for coffee consumption associated with cardia cancer (HR, 1.23; 95% CI, 1.04 to 1.45), and no statistically significant heterogeneity across studies (I^2^ = 36.4, *P* = 0.207).

**Table 2:** Subgroup meta-analysis for the relationship between coffee consumption and risk of gastric cancer.

					Heterogeneity Test	
Subgroups		N	HR (95% CI)	Pooling Model	I^2^ (%)	*P*
Sex	Men	5	1.14(0.76–1.70)	Random	85.9	0.000
	Women	5	1.07(0.70–1.64)	Random	84.1	0.000
Anatomic location	Cardia	3	1.23(1.04–1.45)	Fixed	36.4	0.207
	Non-cardia	3	0.90(0.77–1.04)	Fixed	43.7	0.169
Duration of follow-up	<10 years	2	1.04(0.79–1.36)	Random	60.1	0.113
	≥10 years	7	1.06(0.84–1.35)	Random	77.5	0.000
Place of residence	Asia	2	0.86(0.72–1.02)	Fixed	0.0	0.887
	USA	3	1.35(1.02–1.81)	Fixed	49.2	0.140
	Europe	4	0.98(0.71–1.34)	Random	79.8	0.002
Race	Japanese	3	1.27(0.79–2.06)	Random	75.0	0.018
	Swiss	2	1.19(0.59–2.40)	Random	78.5	0.031
Confounders adjusted for	Alcohol	6	1.09(0.87–1.37)	Random	80.6	0.000
	Smoking	7	0.97(0.81–1.15)	Random	71.6	0.002
	Above two and dietary factors	3	1.02(0.87–1.18)	Random	47.5	0.149

### Dose-response analysis of coffee consumption and the risk of gastric cancer

**Fig 4:**
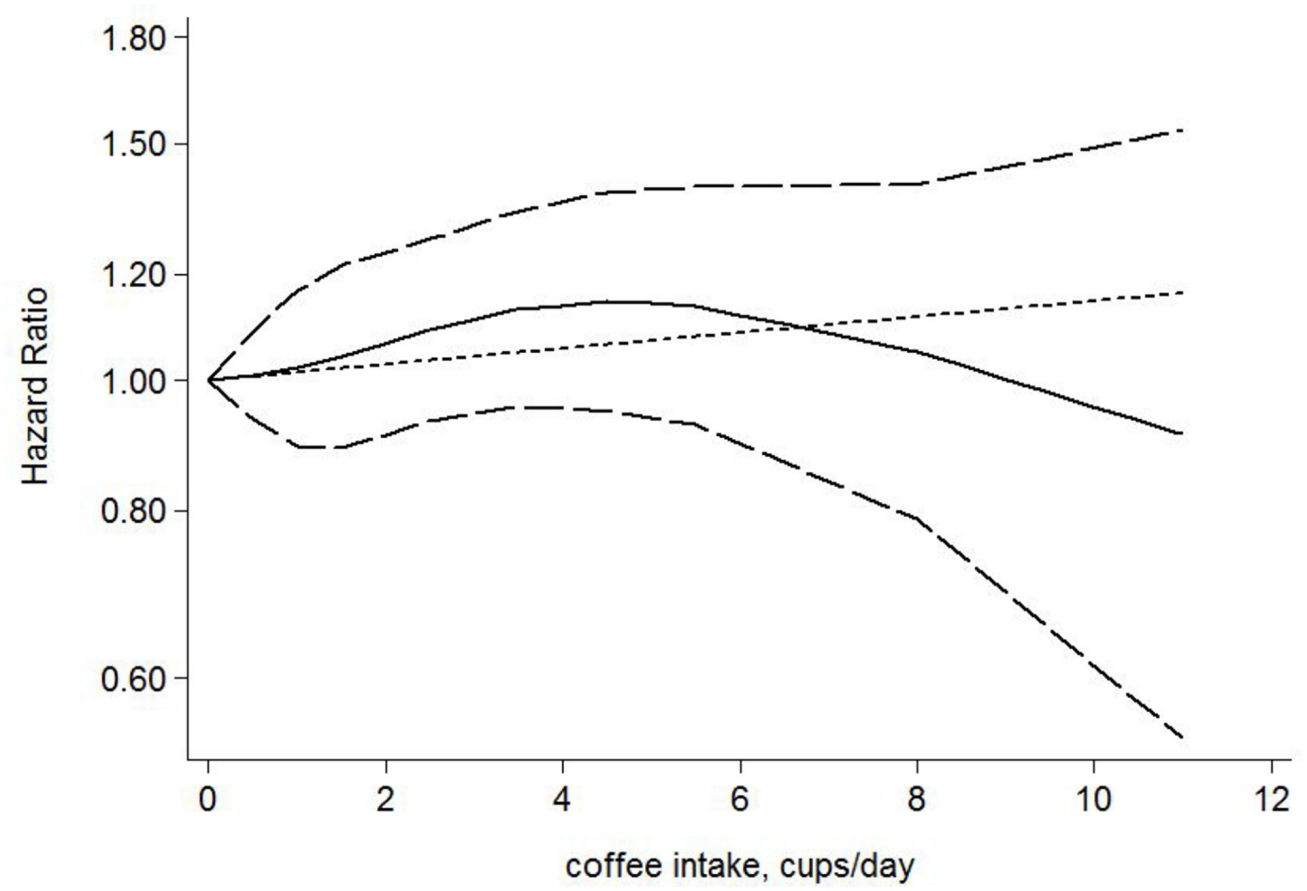
The dose-response analysis between coffee consumption and the risk of gastric cancer. Cups of coffee consumed were modeled with a multivariate fixed-effects dose-response model. The solid line and the long dashed line represent the estimated HR and its 95% CI for the nonlinear relationship. The short dashed line represents the linear relationship.

Five studies [13,14,16–18] were included in our dose-response analysis. A fixed-effects model was applied with no evidence of heterogeneity (Q = 20.98, P = 0.102). No significant association was observed between cups of coffee consumed and the risk of gastric cancer (P for linearity trend or non-linearity > 0.05) (Fig 4). An increment of 1 cup per day was not significantly associated with the risk of gastric cancer (HR, 1.01; 95% CI, 0.98 to 1.05).

### Publication bias

**Fig 5:**
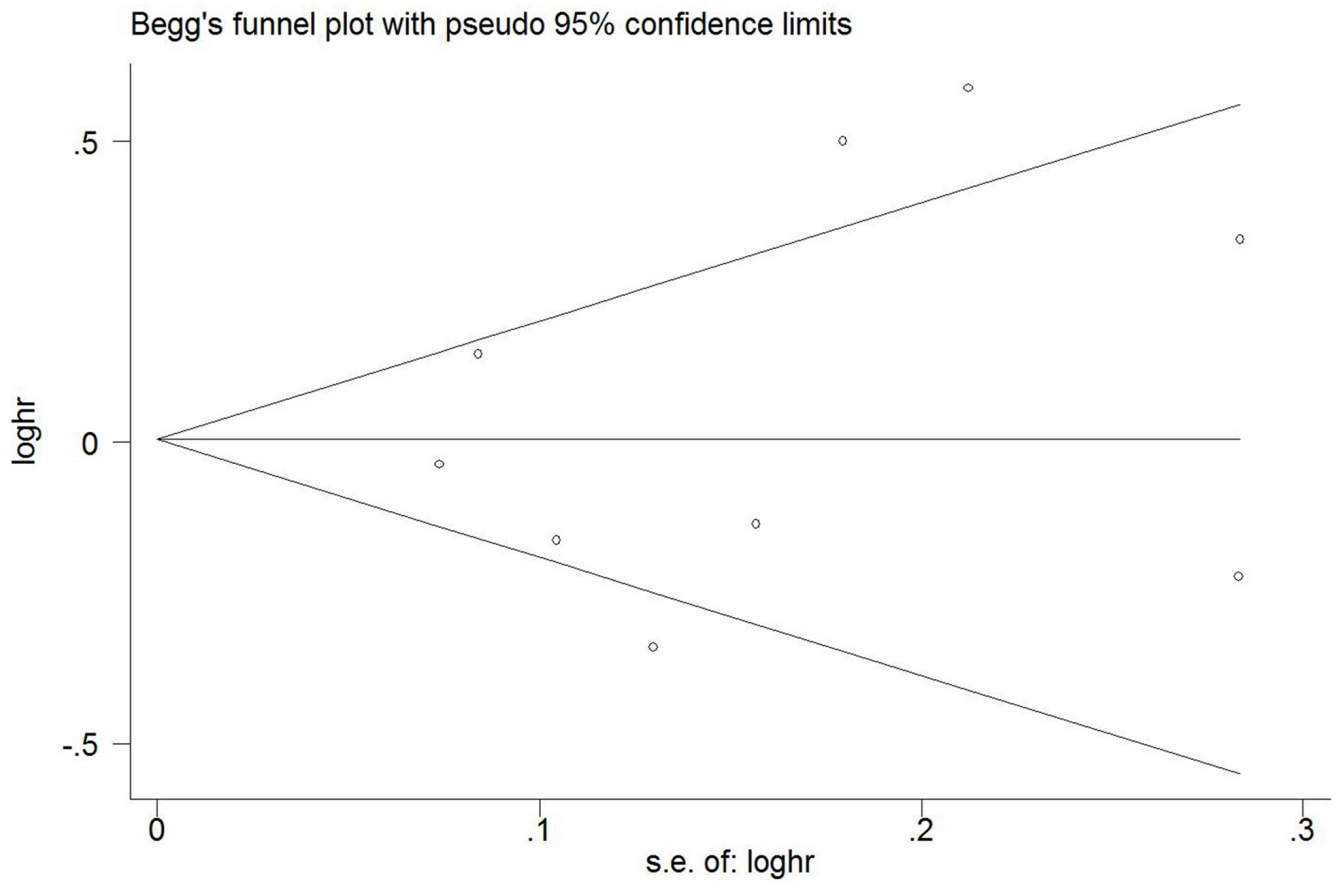
Funnel plot. Begg’s funnel plot with 95% confidence limits assessing publication bias for the association between coffee consumption and the risk of gastric cancer.

The *P* value of Egger’s test and Begg’s test was 0.602 for “regular coffee drinker” versus “seldom drink coffee” categories, which suggested no publication bias. A Begg’s funnel plot was also used to examine publication bias (Fig 5), and no significant publication bias was observed.

## Discussion

In the present meta-analysis, we found that coffee intake was not significantly associated with overall gastric cancer risk. Because there was significant heterogeneity among the studies, a sensitivity analysis was conducted to explore possible explanations for heterogeneity. After deleting the three studies that were the main origin of heterogeneity, the summary HR ranged from 1.05 (95% CI, 0.88 to 1.25) to 0.99 (95% CI, 0.91 to 1.08), which suggested that the association remained insignificant and our findings were reliable and robust. When we further explored the causes of heterogeneity by subgroup analysis, the differences of anatomic location, place of residence, and adjusted confounders might have contributed to the heterogeneity among the studies. From subgroup analysis by anatomic location, we concluded that coffee consumption was a risk factor for cardia cancer but was not associated with the risk of gastric non-cardia cancer, which does not contradict the previous conclusion. The subgroup analysis by place of residence showed that Asia and USA presented low heterogeneity and the pooled result from studies of USA showed a significant positive association. Given that USA is one of countries with the highest proportion of cardia cancers [2], it is not surprising that a significant positive association between coffee consumption and the risk of gastric cancer was observed in USA. The subgroup analysis by adjusted confounders, including smoking, alcohol drinking and dietary factors, also presented low heterogeneity, but the association remained insignificant, which confirmed our findings.

Compared with a meta-analysis focusing only on comparing the extreme categories of coffee consumption (high versus low), a dose-response meta-analysis can accurately assess the relationship between the amount of coffee consumed and the risk of gastric cancer. Therefore, we further conducted a dose-response analysis to verify our previous conclusion. Similar results were observed in the dose-response analysis; there was no significant association between cups of coffee consumed and the risk of gastric cancer.

Furthermore, we analyzed and summarized the special studies included in the systematic review but excluded from the meta-analysis. There were three special studies with reference categories of ≤ 2 cups/day or ≤ 3 cups/day. The effect sizes of these studies were not pooled into our meta-analysis because the reference categories could not be unified. The study by van Loon et al. [24] was not consistent with our findings; it suggested that coffee intake might increase the risk of gastric cancer. Possible reasons for this discrepancy include the relatively small sample size in that study and the use of a different reference category.

Coffee is a complex mixture that contains many chemical substances. There are some potential mechanisms through which coffee may modulate the risk of gastric cancer. For example, coffee contains some phenolic compounds such as chlorogenic acid and caffeic acid, which have anti-cancer properties [25–27], and coffee can stimulate the release of gastrin [28], which may be involved in the development of gastric cancer [29,30]. It is worth mentioning that there are different mechanisms for cardia cancer and gastric non-cardia cancer. Helicobacter pylori infection is a key determinant of gastric non-cardia cancer [31], but is not associated with cardia cancer [32]. Some phenolic compounds in coffee, such as chlorogenic acid, cafestol and kahweol, can decrease oxidative stress in stomach mucosa induced by helicobacter pylori[33–36], by which coffee may reduce the risk of gastric non-cardia cancer [37]. The exact mechanism that how coffee impact cardia cancer is not yet clear. Further research is required to explain the association between coffee consumption and gastric cancer.

A recent similar meta-analysis included papers published before June 2014 and was published in September 2014 [4]. This meta-analysis pooled the relative risks comparing the highest versus lowest categories of coffee intake to obtain a summary estimate. There was a great difference in borderline of the highest and lowest categories of coffee intake among included studies, which could lead to methodological heterogeneity. A second serious error of the previous meta-analysis is that studies with different outcome measures were included in their meta-analysis. For example, the Khan et al. Study [38] used mortality as the outcome measure, which are different from the other included studies using morbidity as the outcome measure. Therefore, their results are likely to be affected by clinical heterogeneity. Those misclassifications of outcome measures and coffee consumption categories made their conclusions be questioned. We searched literatures before December 2014 and included a new study. Four prospective cohort studies included in the previous meta-analysis were excluded from our meta-analysis because the outcome measure was not incidence of gastric cancer and the reference categories of those studies were not consistent with that of our study. Our meta-analysis also has many advantages compared to the previous study. First, some studies have suggested that the relationship between coffee consumption and gastric cancer is related to anatomic location. In our study, meta-analyses were performed separately for cardia cancer and gastric non-cardia cancer. Second, consistent results from the sensitivity analysis, subgroup analysis and dose-response analysis indicated that our results were robust and reliable. Finally, publication bias is likely to have appreciably influenced their results. In our meta-analysis, more stringent inclusion criteria materially change the *P* values from Egger’s and Begg’s tests, together with the absence of significant asymmetry in the funnel plot, indicate no evidence of publication bias.

Despite these advantages, our study still has some limitations. First, the adjusted confounding factors were diverse among the included studies. In some studies, potentially important confounding factors, such as age, race, alcohol intake, tea consumption and smoking, were not adjusted. For example, the Nomura et al. study [20] only controlled for age. Second, measure methods for coffee consumption were not uniform. In the included studies, the number of cups, times or millilitres per day was used to assess the level of coffee consumption and there were differences in the coffee cup sizes. Third, there were differences in the caffeine content and coffee processing methods. Fourth, there were differences in collection instruments of coffee consumption data, such as food frequency questionnaires, diet habit questionnaires and dietary recall history, which might have contributed to the heterogeneity among the studies. Finally, based on existing data, we did not discuss whether the risk of gastric cancer increased as the duration of coffee consumption increased because most studies did not provide the duration of coffee consumption.

In summary, the results from this meta-analysis of prospective cohort studies demonstrated that coffee consumption was not associated with overall gastric cancer risk. Our study confirmed the conclusions from the previous meta-analysis. In addition, we have made an important discovery that coffee consumption might be a risk factor for gastric cardia cancer. Future prospective studies that include information on coffee consumption throughout life, as well as the type of coffee consumed (e.g., caffeinated vs decaffeinated) and the anatomical locations of gastric cancer, and studies that consider potential confounders (e.g., *H*. *pylori* infection and precancerous gastric cancer lesions) are required to confirm our conclusions.

## Supporting Information

S1 Checklist PRISMA checklist. (PDF)

S1 Fig Forest plot of the remained 6 studies after sensitivity analysis. A forest plot for the study-specific regularly versus seldom coffee drinking categories after excluding three studies to reduce heterogeneity. The combined hazard ratio (HR) and 95% confidence intervals (CI) was calculated using the fixed-effects model.
(TIF)
