# Supplementary figures and images for: Effect of Coffee Consumption on the Risk of Gastric Cancer: A Systematic Review and Meta-Analysis of Prospective Cohort Studies
Source: PLoS One. 2015 May 29;10(5):e0128501. doi: 10.1371/journal.pone.0128501 (PMC4449182; doi:10.1371/journal.pone.0128501)

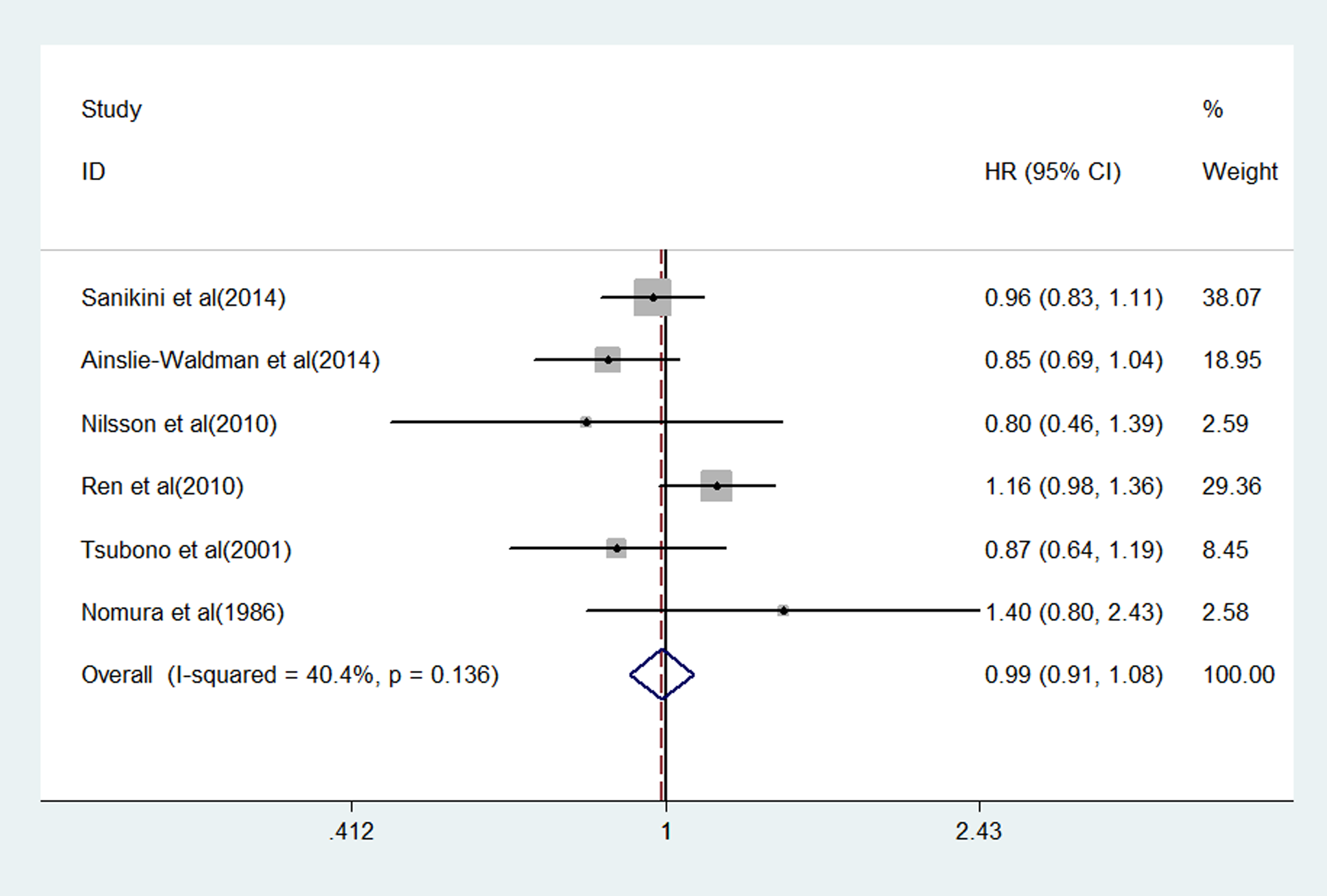

Supplement: S1 Fig — A forest plot for the study-specific regularly versus seldom coffee drinking categories after excluding three studies to reduce heterogeneity. The combined hazard ratio (HR) and 95% confidence intervals (CI) was calculated using the fixed-effects model. (TIF) [file pone.0128501.s002.tif]
